# Development of a nutritional screening and assessment indicator system for patients with esophageal cancer in China: Findings from the Delphi method

**DOI:** 10.1002/cam4.6703

**Published:** 2023-11-21

**Authors:** Jingjing Shang, Wen Dong, Peipei Huang, Yidan Sun, Yuxin He, Hui Li, Shengwu Liao, Mei Li

**Affiliations:** ^1^ Department of Thoracic Surgery Southern Medical University Nanfang Hospital Guangzhou China; ^2^ School of Nursing Southern Medical University Guangzhou China; ^3^ Department of Health Management Southern Medical University Nanfang Hospital Guangzhou China

**Keywords:** Delphi method, esophageal cancer, index system, nutritional assessment, nutritional screening

## Abstract

**Background:**

In China, individuals diagnosed with esophageal cancer are confronted with an elevated risk of nutritional inadequacy or malnutrition throughout the course of their disease, a condition that contributes to various adverse clinical outcomes. A vast corpus of data are burgeoning at an unprecedented rate, primarily due to the revolutionary growth of digitalization technologies and artificial intelligence, notably within the domains of health care and medicine. The purpose of this investigation is to initiate the development of a nutritional screening and assessment indicator framework for patients with esophageal cancer within the Chinese context. We seek to furnish an instrumental reference to facilitate preparations for the forthcoming era of advanced, “deep,” evidence‐based medicine.

**Methods:**

An integrative methodology was employed to forge the preliminary draft of the nutritional screening and assessment indicator system for preoperative patients with esophageal cancer. This encompassed a rigorous literature survey, in‐depth clinical practice investigation, and the facilitation of expert panel discussions. Thereafter, two iterative consultation phases were conducted using the Delphi method in China. The analytic hierarchy process was deployed to ascertain the weighting of each index within the definitive evaluation indicator system.

**Results:**

The effective response rates for the dual rounds of expert consultation were 91.7% and 86.4%, with commensurate authority coefficients of 0.97 and 0.91. The Kendall harmony coefficients were ascertained to be 0.19 and 0.14 (*p* < 0.01), respectively. The culminating nutritional screening and assessment indicator system for patients with esophageal cancer comprised 5 primary‐level indicators and 38 secondary‐level indicators.

**Conclusions:**

The nutritional screening and assessment indicator system contrived for patients with esophageal cancer is underpinned by cogent theoretical principles, leverages an astute research methodology, and manifests dependable outcomes. This system may be appositely utilized as a meaningful reference for the nutritional screening and assessment process in patients afflicted with esophageal cancer.

## 1 INTRODUCTION

China perennially constitutes a high‐incidence region for esophageal cancer on a global scale, harboring the majority of esophageal cancer cases across Asia.^1^ Both the incidence and mortality rates pertinent to esophageal cancer within the Chinese demographic remain elevated.^2^ Over the preceding years, concomitant with the precipitous advancement of technology, methodologies for the treatment of esophageal cancer have witnessed substantial progression. Techniques encompassing surgical interventions, neoadjuvant therapies, and adjuvant therapies have markedly enhanced patient prognosis and survival rates.^3^, ^4^, ^5^ Nevertheless, a multitude of factors, inclusive of the singular location of the lesion, tumor metabolic anomalies, augmented basal metabolism, chronic inflammatory response, tumor treatment, advanced age of onset, late‐stage diagnosis, suboptimal prognosis, and elevated recurrence rates have engendered a profound connection between esophageal cancer patients and their nutritional status.^6^, ^7^, ^8^, ^9^

A predilection for malnutrition is accentuated in cancer patients, with global studies delineating the prevalence of malnutrition to oscillate between 20% and 80%, displaying heterogeneity contingent upon variables such as patient age, tumor classification, tumor locale, disease trajectory, and antitumor therapeutic regimens, inter alia.^10^, ^11^ Individuals afflicted with digestive tract tumors manifest a heightened incidence of malnutrition in comparison to other cancer forms, especially those with upper gastrointestinal tract tumors, which may escalate to an alarming 80%.^12^ Esophageal cancer patients, commonly diagnosed at advanced stages due to frequently unobtrusive early indications, experience malnutrition upon admission in an estimated range of 40%–60%.^6^, ^9^ This underscores that esophageal cancer patients are confronted with an augmented nutritional risk and malnutrition incidence throughout their disease course, concomitant with a significant prevalence of deleterious clinical sequela, such as intensified infection‐related complications, protracted hospitalizations, and escalated health care expenditures.^13^ In alignment with this, the Chinese Anti‐Cancer Association's Committee of Oncology Nutrition, in collaboration with the Chinese Society for Parenteral and Enteral Nutrition, promulgated the “Nutritional Therapy Guidelines for Esophageal Cancer Patients (2022),” advocating the execution of nutritional risk screening for all diagnosed esophageal cancer patients. Should nutritional risk be discerned, subsequent nutritional status evaluations must be undertaken to assess the presence and gravity of malnutrition, thereby orchestrating pertinent nutritional interventions and management strategies, with an aspiration toward appreciably ameliorating patients' nutritional well‐being and quality of life.^14^

Malnutrition in patients with esophageal cancer is predominantly classified under the umbrella of tumor‐related malnutrition, a specialized form of chronic disease‐associated malnutrition accompanied by inflammation.^13^, ^15^, ^16^ Distinct from malnutrition arising solely from inadequate food consumption or anorexia, such as starvation‐induced malnutrition, tumor‐related malnutrition places emphasis on the intricate pathophysiological processes associated with the tumor itself. This category of malnutrition is characterized by a diminished nutrient intake and metabolic disturbances, both of which are elicited by chronic inflammation attributable to the neoplasm. The resulting energy imbalance manifests in alterations to body composition, compromised functionality, and clinical manifestations such as anorexia and weight loss, collectively referred to as cachexia.^17^, ^18^ Cachexia is not an uncommon occurrence within the context of malignancies and certain chronic ailments, including heart failure, kidney failure, and chronic obstructive pulmonary disease, exhibiting prevalence rates that vary between 20% and 80%.^19^, ^20^ Specifically, among individuals with esophageal cancer, the prevalence of cachexia can be as high as 80%, rendering it an essential consideration in the preoperative evaluation and subsequent postoperative recovery phases.^21^ Notwithstanding its undisputed clinical relevance, the precise pathophysiological underpinning of cachexia remains only partially elucidated. The constellation of factors contributing to cachexia encompasses a disrupted energy equilibrium, an asymmetry in the secretion of hormones and cytokines by both host and tumor cells, and perturbations in the regulation of hypothalamic energy expenditure. These elements together wield a substantial influence on the nutritional well‐being and overall health of the afflicted patients.^22^

The intricate interplay between neoplasms, the tumor microenvironment, and diverse organ systems catalyzes the manifestation of tumor‐associated malnutrition within cancer patients.^23^ The tumor microenvironment (TME)—composed of cancer cells, assorted stromal cell populations (such as infiltrating immune cells, neutrophils, fibroblasts, and adipocytes), extracellular matrix constituents, soluble factors, and signaling molecules—exerts an instrumental role in inciting systemic inflammation and augmented oxidative stress in cancer patients.^24^ Systemic inflammation serves as a fundamental facet in the pathophysiology of tumor‐associated malnutrition (cancer cachexia), and its significance is accentuated by global leadership initiative on malnutrition (GLIM) on malnutrition diagnostic criteria.^25^, ^26^ Systemic inflammation materializes as an indiscriminate response to tumor hypoxia/necrosis or localized tissue damage, epitomizing the host's immunological reaction to the tumor presence. An imbalance between pro‐inflammatory cytokines and anti‐inflammatory cytokines, modulated by inflammatory cells derived from the host and cancer cells (such as macrophages, neutrophils, fibroblasts, lymphocytes, and monocytes), culminates in the synthesis of pro‐inflammatory cytokines (such as interleukin‐1, interleukin‐6, interferon‐gamma, tumor necrosis factor‐α, TGF‐β, etc.). These factors, either in isolation or conjunction, expedite the deterioration of the tumor microenvironment, further propelling systemic inflammation, precipitating immune dysregulation, and engendering metabolic aberrations across multiple organ systems.^27^

In the context wherein organs and neoplasms are at a comparatively balanced stage, the emergence of anorexia, characterized by diminished dietary intake, manifests as an initial symptomatology. The etiological mechanism governing anorexia is multifactorial, encompassing cellular components secreted by both the neoplasm and host, which predominantly contribute to this phenomenon. The hypothalamus functions as the pivotal modulator of energy equilibrium, exerting control over both food consumption and the broader scope of energy balance.^28^ Cellular factors, including interleukin‐6 (IL‐6) and tumor necrosis factor‐alpha (TNF‐α), or endocrine hormones such as leptin and growth hormone‐releasing peptides, interact with receptors located on hypothalamic neurons. These receptors interpret them as molecular harbingers of pathological conditions. Hypothalamic neurons receive these signals and enhance their impact, thus modulating the functionality of neurons engaged in appetite and metabolic pathways, such as those involving hypothalamic neuropeptide Y, Agouti‐related protein neurons, and pro‐opiomelanocortin (POMC). Through the intricately connected hypothalamus–pituitary–adrenal axis, these interactions catalyze the catabolism of proteins and fats within skeletal muscles, adipose tissue, and other tissues. This cascade gives rise to an array of illness‐related behaviors, comprising anorexia, weight attrition, and muscle atrophy. Cellular factors synthesized by neoplastic cells instigate the hypothalamic neurons to induce POMC expression, consequently exacerbating the stimulation of the central melanocortin system.^21^ Leptin, a protein discharged by subcutaneous adipose tissue, serves to regulate appetite, augment energy expenditure, and preserve energy equilibrium by mediating the hypothalamic pathway that fosters appetite. Diminished leptin levels incite pathways that promote appetite, augmenting neuropeptide Y activity, thereby encouraging food intake. Conversely, elevated leptin levels overstimulate the anorexigenic pathway, culminating in the suppression of food intake and a surge in energy expenditure. Diakowska et al. expansive study on esophageal carcinoma posits leptin as a prospective predictor for cachexia.^29^ Growth differentiation factor 15 (GDF‐15), a constituent of the transforming growth factor‐beta superfamily, exhibits dual roles. It not only exerts a direct influence on the brainstem's feeding center, eliciting anorexia, but also modifies the body's metabolic pathways, resulting in muscle and fat depletion. Additional molecules, such as TNF‐α, ghrelin, and PTHrP, facilitate the regulation of appetite and dietary intake via diverse mechanisms.^21^, ^23^, ^28^

During the nascent stages of cancer‐related malnutrition, referred to as cancer cachexia, an escalation in visceral organ and tumor mass represents one variant of the body's increased resting energy expenditure. The liver's seminal role in energy metabolism encompasses the handling of the body's three principal fuel substrates: glucose, fats, and proteins. It orchestrates both the storage and liberation of energy. Within cachexia patients, phenomena such as impaired glucose tolerance and insulin resistance are frequently observed, with insulin resistance amplifying concomitant with disease progression. Insulin resistance predominately propels hepatic gluconeogenesis and culminates in diminished insulin receptor tyrosine kinase activity, abated binding of insulin and insulin‐like growth factor‐1 to receptors, and compromised signaling via the PI3K/AKT/mTOR pathway. This cascade of events restrains protein synthesis and fosters the degradation of muscle tissue. The resultant release of amino acids into the bloodstream further augments tumor cells' utilization of aerobic glycolysis metabolic pathways, thereby enhancing tumor growth and hastening energy depletion.^23^

Within the patient population afflicted by cancer‐related malnutrition (cancer cachexia), the onset of lipolysis is observed to precede muscular atrophy. A cohort study conducted in Sweden, evaluating the alterations in body composition among patients diagnosed with gastrointestinal solid neoplasms, discerned that the depletion of adipose tissue occurred at an accelerated rate compared to the loss of lean body mass in individuals experiencing progressive cachexia.^30^ This observation complements the previously described phenomena of impaired glucose tolerance and insulin resistance, which can facilitate adipose catabolism. Furthermore, certain pro‐inflammatory cytokines, namely tumor necrosis factor‐alpha (TNF‐α) and the tumor‐derived factor zinc‐alpha2‐glycoprotein (ZAG), are implicated in these processes. Specifically, TNF‐α mediates adipose breakdown by suppressing the enzymatic activity of lipases and the glucose transporter 4 (GLUT4), consequently inhibiting fat synthesis. Moreover, TNF‐α possesses the ability to instigate the c‐Jun N‐terminal kinase (JNK) MAP (mitogen‐activated protein) pathway, while simultaneously curtailing the differentiation of preadipocytes. Acting as a lipolytic effector, ZAG induces lipid utilization and augments fat oxidation within brown adipose tissue, thereby elevating overall energy expenditure. It is noteworthy that adipose tissue can synthesize factors that instigate systemic inflammation, such as TNF‐α and interleukin‐6 (IL‐6), and that an excessive secretion of these elements may catalyze lipolysis.^28^

Concurrently, skeletal muscle depletion emerges as a salient characteristic within tumor‐related malnutrition (cancer cachexia).^19^ This is principally attributable to the concomitant reduction in protein synthesis and amplification of protein catabolism, culminating in the loss of muscular tissue in afflicted patients.^31^ The catabolic metabolism of skeletal muscle protein is orchestrated by three principal proteolytic systems: the ubiquitin‐proteasome pathway, the calcium‐activated mechanism, and the autophagy‐lysosome pathway. Protein degradation‐inducing factors (PIFs) accentuate protein catabolism by stimulating the ubiquitin‐proteasome pathway (UPP). They modify protein stability through the enhancement of ubiquitination and elevate the mRNA levels of proteasome subunits, thereby facilitating protein degradation. However, the precise functioning of this mechanism continues to be an area of contention within human research. Additionally, PIFs have the capacity to inhibit protein synthesis via the activation of RNA‐dependent protein kinase. Supplementary investigations have unveiled that specific cytokines, encompassing transforming growth factor‐beta, IL‐1, IL‐6, myostatin, and activins, can actuate the metabolic breakdown of skeletal muscle.^32^

## 2 MATERIALS AND METHODS

The diagnostic evaluation of malnutrition necessitates an integrative examination of multifaceted nutrition‐related metrics, encompassing clinical history, body mass index (BMI), longitudinal weight loss trajectory, dietary status, blood biochemistry profile, body composition, clinical symptomatic manifestations, and psychological and social support systems, among others. The inherent complexity of this evaluation is compounded by regional variations in geographical milieu, disease manifestation, demographic attributes, and concomitant disparities in economic conditions, resource limitations, equipment accessibility, and professional expertise. Consequently, there is a conspicuous absence of a unified gold standard for the diagnosis of malnutrition, thereby further convoluting the selection process of nutrition screening or assessment indices (indicator sets), evaluative tools, measurement techniques, or equipment employed by clinical health care practitioners within specific clinical environments. Such a nuanced process mandates meticulous scrutiny of the overarching context.

Internationally, the standardization of nutritional diagnostic procedures and therapeutic interventions is delineated into three cardinal stages: “Nutritional screening ‐ Nutritional assessment ‐ Nutritional intervention.”^33^ Among the plethora of methodologies, the Nutritional Risk Screening 2002 (NRS‐2002) has emerged as the most widely utilized screening tool. Endorsed by eminent organizations such as the American Society for Parenteral and Enteral Nutrition (ASPEN), the European Society for Clinical Nutrition and Metabolism (ESPEN), and the Chinese Society for Parenteral and Enteral Nutrition (CSPEN), it is employed to gauge nutritional risk within the first 48 h of a patient's hospital admission.^34^, ^35^, ^36^ The realm of nutritional assessment (or diagnosis) has engendered some debate and divergence of opinion. The American Dietetic Association (ADA) and the Committee on Nutrition and Supportive Treatment for Cancer of China Anti‐Cancer Association (CSONSC) advocate the use of the Patient‐Generated Subjective Global Assessment (PG‐SGA) to evaluate nutritional malnutrition specifically in oncology patients. In contrast, ASPEN, ESPEN, and CSPEN withhold recommendation for the utilization of the Subjective Global Assessment (SGA) or PG‐SGA.^37^, ^38^, ^39^ In 2018, ASPEN and ESPEN collaboratively promulgated the Global Leadership Initiative on Malnutrition (GLIM) consensus, with the overarching aim of fostering a universal accord on the diagnosis of clinical malnutrition. Despite these concerted efforts, conventional nutritional screening or assessment instruments, architected for general patient populations, frequently exhibit shortcomings in comprehensively addressing the particularized nutritional exigencies and requisites of diverse patient cohorts, owing to their constrained capabilities. In contradistinction, the present research endeavors to devise a nutrition screening and assessment indicator paradigm singularly tailored for patients afflicted with esophageal cancer. This constitutes a novel, incisive strategy that specifically targets the elevated incidence of nutritional complications within this specialized patient demographic. Furthermore, this approach is characterized by a systematic, modular, and multidimensional architecture that amalgamates a diverse array of nutritional items, indicators, and parameters gleaned from various preexisting nutrition screening and assessment scales, transcending the confines of any singular scale and concurrently accounting for nutrition (malnutrition) risk determinants.

The inaugural framework for nutrition screening and assessment for esophageal cancer patients is segmented into five discrete modules (dimensions): clinical history, dietary survey, nutrition impact symptoms, anthropometric evaluations, and blood biochemistry markers. The overarching objective of this endeavor is to meticulously identify the most propitious nutritional indicators and evaluative methodologies tailored to specific clinical scenarios, thereby furnishing more precise nutrition screening and assessment protocols to facilitate clinical nutrition diagnosis, intervention, and oversight. This approach aspires to optimize the enhancement of the patients' nutritional status. Consequently, this index system will erect a theoretical framework and contribute empirical evidence to steer future explorations in this field. Such an approach is envisaged to prepare the way for the next generation of “deep” evidence‐based medicine—a paradigm poised to profoundly synthesize and integrate the entire compendium of accessible data.^40^

In furtherance of this objective, we have methodically constructed a nutritional screening and assessment indicator apparatus for preoperative patients diagnosed with esophageal cancer. This has been achieved through a scrupulous synthesis of the existing literature and the application of the rigorous Delphi method. The foundational theories and principles that form the bedrock of this indicator system were extracted from an exhaustive review of pertinent literature, complemented by the invaluable insights of stakeholders, gleaned through the cooperative endeavors of the research team. Subsequently, the system was subjected to additional refinement through the synergistic application of both the Delphi process and the analytic hierarchy process (AHP).

### 2.1 Development of the framework for constructing a nutritional screening and assessment indicator system for patients with esophageal cancer

#### 2.1.1 Literature source and retrieval method

An exhaustive examination of extant nutritional screening and assessment indicators was undertaken with the objective of identifying potential indices and evaluative methodologies pertinent to the formulation of a specialized system. A multifaceted search encompassing both English and Chinese databases, including but not limited to PubMed, Science Direct, Web of Science, Embase, Chinese Biomedical Literature Database, China National Knowledge Infrastructure, China Science and Technology Journal Database, and Wanfang, was conducted to retrieve germane articles on nutritional screening and assessment indicators, tracing back to the inception of these databases.

The search methodology implemented an amalgamation of MeSH/Emtree terms and free terms, with keywords such as “neoplasms,” “carcinoma,” “tumor*,” “cancer*,” “nutrition assessment,” “nutrition surveys,” “nutritional status,” “nutrition disorders,” “malnutrition,” “protein‐energy malnutrition,” “nutrition* deficiency*,” “nutrition* screening*,” “nutrition* risk,” “malnutrition* risk,” “undernutrition,” “malnourishment*,” and the like. These terms were utilized individually or in conjunction with “OR” and/or “AND” logical operators. A supplementary snowball method was exercised, entailing a meticulous examination of the original studies' references to identify additional pertinent research. Moreover, the relevant contents from nutritional curricula and authoritative textbooks were referenced to augment the search.

The amassed data were diligently organized, classified, and subjected to rigorous content juxtaposition and analysis. Through collaborative discourse, the research team achieved a consensus on shared insights, culminating in the synthesis of salient indications instrumental to the provisional construction of a nutritional screening and assessment indicator system uniquely adapted for preoperative patients diagnosed with esophageal cancer.

### 2.2 Delphi process

The Delphi method was utilized to scrutinize the results stemming from the literature review. As a qualitative research approach predominantly employed within the social sciences, the Delphi method was originally conceived by the Rand Corporation during the 1950s and subsequently amplified by Dalkey and Helmer in 1963. It encompasses a sequence of iterative cycles of expert dialogues concentrated on arriving at a consensus regarding a specific subject matter. Within this paradigm, each expert's perspective is accorded equal significance, underpinned by the guarantee of anonymous feedback, thereby mitigating any undue influence from individual characteristics, such as self‐assurance, political predispositions, or social standing.^41^, ^42^, ^43^, ^44^

### 2.3 Expert panel selection and recruitment

The criteria for inclusion encompassed the following considerations: (1) attainment of a Bachelor's degree or superior academic qualifications; (2) inclusion of clinicians and nursing experts bearing the deputy senior title or higher, in addition to clinical nutritional experts possessing an intermediate title or above; (3) clinicians and nursing experts must have accrued over a decade of professional experience, and clinical nutritional experts must possess a minimum of 5 years of practice; (4) all professionals are expected to manifest an extensive reservoir of theoretical acumen and empirical expertise across a wide spectrum of domains, including but not limited to nutritional assessment, intervention, medical treatments, and nursing care; (5) voluntary engagement in this study, accompanied by the fulfillment of expert consultations, is mandated as a prerequisite.

### 2.4 Data collection process

The execution of the Delphi study was diligently pursued in strict adherence to established protocols. Instruments of inquiry, predominantly in the form of questionnaires, were disseminated to selected experts via electronic mediums such as email or WeChat, with subsequent reminders promulgated through succinct message formats to expedite timely completion. Each iterative phase of the study necessitated the experts' engagement and submission of feedback within a specified biweekly window. Non‐responsiveness within the designated timeframe engendered reminders to stimulate active participation. Successive rounds of solicitation were commenced only upon the comprehensive acquisition of feedback from the constituent experts of the preceding phase. The determination to advance to successive rounds of consultation was predicated upon the synthesized results and was guided by the overarching principle of information saturation. Concomitantly, meticulous precautions were enacted to insulate the Delphi process from undue influence, whether emanating from internal team members or external expert sources.

### 2.5 Design of expert consultation questionnaire

The architectonics of the questionnaire were systematically delineated into quartile sections. The inaugural section embodied an expository missive to the expert constituency, articulating an overview of the research milieu, objectives, present status, and stipulations attendant to the completion of the questionnaire.

The secondary section embodied the nexus of the expert inquiry, evaluating the relative significance (Cij) of individual indicators germane to the nutritional screening and assessment for preoperative patients diagnosed with esophageal cancer. Experts were enjoined to ascribe importance via the utilization of a 5‐point Likert scale methodological approach, the extremities of which were demarcated at 5 points for “very important” and 1 point for “not important.” These ascriptions were contingent upon empirical evidence, exigency, and practicability. Additionally, experts were exhorted to contribute qualitative reflections and recommendations.

The tertiary section was designed to assimilate demographic and professional metrics pertaining to the experts, encompassing variables such as age, gender, professional credentialing, educational lineage, and cumulative tenure in their respective vocations.

The quaternary section was oriented toward an assessment of the professionals' acuity with the proffered indications and the substratum of their judgment. Familiarity (Cs) was quantified on a continuum scale from 0 to 1, with elevated scores symptomatic of heightened familiarity. The scale's polarities were characterized at 1 point for “very familiar” and 0.2 points for “not familiar.” Judgment basis (Ca) necessitated the expert's stratification of the magnitude of influence into discrete classifications, identified as large, medium, or small, anchored by their reflective judgment.

### 2.6 Calculation of the indicator framework

The data analysis necessitated the deployment of designated statistical software, specifically Excel 2016 for the genesis of a structured database, and SPSS 26.0 for the orchestration of rigorous statistical analyses. The duties of data input, verification, and precision analysis were allocated to two designated individuals. Expert consultations transpired across two distinct rounds, wherein experts were tasked with the assessment of the importance (Cij), familiarity (Cs), and judgment basis (Ca) of each constituent indicator within the framework. In scenarios where commentary from two or more experts converged on an identical indicator, further investigation and deliberative discourse were undertaken by the research team, drawing upon both the content of the indicator and the substance of expert feedback. Upon the conclusion of the inaugural round of consultations, the research team undertook subsequent modifications to the indicators. Experts further proffered counsel for the refinement and augmentation of indicators. Notably, new indicators proposed by experts were integrated into the second round of consultation, complemented by an elucidative narrative regarding the adjustments made. The culmination of these processes resulted in the adjustment of the indicator framework, reflective of expert scoring and recommendations. A subsequent round of expert consultation was conducted to solidify the indicator framework for the nutritional screening and assessment of preoperative patients diagnosed with esophageal cancer.

The evaluative criteria were judiciously applied to appraise the indicator framework, utilizing data compiled from the selected expert cohort. These criteria were bifurcated into the following quadrants:

- Positive coefficient: This represented the alacrity of expert engagement, gauged by the actual questionnaire retrieval rate. A recovery rate surpassing 75.0% was emblematic of heightened expert commitment to the research.
- Authority coefficient (Cr): This coefficient symbolized the proficiencies of the experts and was computed as the arithmetic mean of the familiarity degree coefficient (Cs) and the judgment basis coefficient (Ca), as articulated by the equation Cr = (Ca + Cs)/2. A Cr value ≥0.7 was indicative of a pronounced level of expertise, with escalating Cr values denoting an increasing command over the content under consultation.
- Concentration of expert opinions: This segment evaluated the salience of each indicator. Both the mean ($\bar{x}$) and full score percent (*K*) for each index were derived, with the mean being deduced via the formula $\bar{x}=\left(x_{1}+x_{2}+\cdots +x_{n}\right)/n$. The full score percent (*K*) constituted the fraction of maximal scores attained.
- Coordination degree of expert opinions: This was quantified through Kendall's coordination coefficient (Kendall's *W*) and the coefficient of variation (CV), where Kendall's *W* oscillated between 0 and 1, and a more substantial *W* value, coupled with a significant chi‐squared test, signaled superior coordination. The coefficient of variation (CV) was evaluated as CV = standard deviation (SD)/mean value ($\bar{x}$), with a diminished coefficient of variation signifying a more pronounced consensus within the expert community.
- Questionnaire reliability: This was ascertained via Cronbach's alpha coefficient, where a value of ≥0.70 was demonstrative of commendable questionnaire reliability.

In summation, an indicator was earmarked for excision across the two rounds of expert consultation if it fulfilled any of the following benchmarks: (1) mean < 4.0 or full score percent (*K*) < 30%; (2) variation coefficient > 0.25. The Delphi consultation process was deemed to have reached completion if the experts' authority coefficient (Cr) and Kendall's coefficient of concordance (or Kendall's *W*) satisfied the stipulated criteria.

### 2.7 Ethical considerations

In a pursuit to uphold participant confidentiality, all individuals implicated in the study were preserved in anonymity and were meticulously apprised of the research's background, objectives, guidelines for questionnaire administration, anticipated outcomes, and their unequivocal right to abstain from participation. Rigorous protocols were enacted to fortify the confidentiality of the accumulated data. Concomitantly, the Delphi experts rendered voluntary consent, underscoring their informed and deliberate engagement in the investigation prior to the study's initiation.

## 3 RESULTS

### 3.1 Establishment of a framework for the index system

An assiduous synthesis of extensive literary reviews and thorough clinical investigations culminated in the establishment of an embryonic framework for the index system. This nascent construct consisted initially of 5 primary (first‐level) and 42 secondary (second‐level) indexes. During the course of the two‐round Delphi process, the framework underwent methodical refinement. Certain indexes were judiciously excised, incorporated, or adjusted in alignment with established screening criteria. This iterative assessment was further enriched by the panel's exhaustive examination of pertinent literature, academic treatises, and robust scholarly discourse. Consequently, a refined index system emerged, encompassing 5 primary and 38 secondary indexes (refer to Table 2).

### 3.2 Demographic information of experts

The intent of the present study was to articulate an indicator framework for the nutritional screening and assessment of preoperative patients diagnosed with esophageal cancer. To this end, a methodically structured questionnaire was disseminated to experts within a temporal window spanning from August 2022 to January 2023. Of the 24 experts who were extended invitations, 23 acquiesced to partake in the initial round (95.8%), and 19 experts persevered to completion in the second round (86.4%). The dual rounds of inquiry engaged professionals across 14 tertiary comprehensive medical institutions dispersed throughout diverse provinces of China, including but not limited to Guangdong, Beijing, Hubei, Sichuan, Jiangsu, Shanxi, Xinjiang, and Henan. A comprehensive delineation of the demographic characteristics of the experts is cataloged in Table 1.

**TABLE 1:** Descriptive statistics of the experts.

Baseline characteristics		First round (*n* = 22)		Second round (*n* = 18)	
Subject	Option	Quantity	Proportion (%)	Quantity	Proportion (%)
Gender	Male	8	36.4	7	36.8
	Female	14	63.6	12	63.2
Age	30–40 years old	8	36.4	5	26.3
	41–50 years old	7	31.8	8	42.1
	More than 50 years old	7	31.8	6	31.6
Nationality	Chinese	22	100.0	19	100.0
Years of professional life	5–15 years	8	36.4	6	31.6
	16–25 years	4	18.2	3	15.8
	26–35 years	10	45.5	10	52.6
Highest level of education: Bachelor's degree	Bachelor's degree	7	31.8	7	36.8
	Master's degree	7	31.8	4	21.1
	Doctor's degree	8	36.4	8	42.1
Title	The intermediate	7	31.8	6	31.6
	Associate senior	7	31.8	6	31.6
	Senior	8	36.4	7	36.8
Professional field	Clinical oncology	8	36.4	6	31.6
	Clinical oncology nursing	8	36.4	8	42.1
	Clinical nutrition	6	27.2	5	26.3

**TABLE 2:** Final nutritional screening and assessment indicator system for patients with esophageal cancer in China.

Primary indicators	Secondary indicators
1. Case history	1.1 Age
	1.2 Clinical stage
	1.3 Admission symptom score
	1.4 Accepted treatment strategies
	1.5 Performance status
2. Dietary surveys	2.1 The number of meals per day
	2.2 Whether food intake is regularity
	2.3 Food character
	2.4 Reduction considerably in food intake
	2.5 Daily dietary energy intake (valuation)
	2.6 Daily dietary protein intake (valuation)
3. Nutrition impact symptoms	3.1 Decreased appetite
	3.2 Eating difficulty/dysphagia/Swallowing dysfunction
	3.3 Choking or coughing while drinking water (or liquid food)
	3.4 Foreign‐body sensation in the chest
	3.5 Burning sensation in the chest
	3.6 Chest pain
	3.7 Prolonged feeding process
	3.8 Feel full quickly
	3.9 Nausea
	3.10 Vomiting
	3.11 Diarrhea
	3.12 Constipation
4. Anthropometric parameters and history	4.1 Body mass index (BMI)
	4.2 Unintended weight loss
	4.3 Dominant hand grip strength
	4.4 Triceps skinfold thickness
	4.5 Arm muscle circumference
	4.6 Calf circumference
5. Blood‐biochemical indicators	5.1 Serum albumin
	5.2 Serum prealbumin
	5.3 Transferrin
	5.4 Retinol‐binding protein
	5.5 Total lymphatic count
	5.6 Hemoglobin
	5.7 Total protein
	5.8 C‐reactive protein
	5.9 Serum total cholesterol

**TABLE 3:** Weight values of Delphi consultation indicators.

Index type		Weight	Combined weight
Primary indicators	Secondary indicators		
1. Case history		0.19	0.97
	1.1 Age	0.25	1.30
	1.2 Clinical stage	0.18	0.91
	1.3 Admission symptom score	0.36	1.85
	1.4 Accepted treatment strategies	0.09	0.44
	1.5 Performance status	0.12	0.63
2. Dietary surveys		0.32	1.68
	2.1 The number of meals per day	0.09	0.54
	2.2 Whether food intake is regularity	0.09	0.54
	2.3 Food character	0.14	0.83
	2.4 Reduction in food intake considerably	0.31	1.87
	2.5 Daily dietary energy intake (valuation)	0.19	1.16
	2.6 Daily dietary protein intake (valuation)	0.19	1.16
3. Nutrition impact symptoms		0.11	0.55
	3.1 Decreased appetite	0.11	1.37
	3.2 Eating difficulty/dysphagia/swallowing dysfunction	0.18	2.22
	3.3 Choking or coughing while drinking water (or liquid food)	0.14	1.76
	3.4 Foreign‐body sensation in the chest	0.07	0.85
	3.5 Burning sensation in the chest	0.05	0.60
	3.6 Chest pain	0.07	0.85
	3.7 Prolonged feeding process	0.04	0.47
	3.8 Feel full quickly	0.05	0.60
	3.9 Nausea	0.11	1.37
	3.10 Vomiting	0.09	1.13
	3.11 Diarrhea	0.07	0.85
	3.12 Constipation	0.03	0.36
4. Anthropometric parameters and history		0.24	1.28
	4.1 Body mass index (BMI)	0.20	1.23
	4.2 Unintended weight loss	0.29	1.78
	4.3 Dominant hand grip strength	0.12	0.72
	4.4 Triceps skinfold thickness	0.20	1.23
	4.5 Arm muscle circumference	0.12	0.72
	4.6 Calf circumference	0.07	0.42
5. Blood‐biochemical indicators		0.14	0.73
	5.1 Serum albumin	0.23	2.21
	5.2 Serum prealbumin	0.23	2.21
	5.3 Transferrin	0.13	1.24
	5.4 Retinol‐binding protein	0.05	0.44
	5.5 Total lymphatic count	0.07	0.67
	5.6 Hemoglobin	0.09	0.88
	5.7 Total protein	0.09	0.88
	5.8 C‐reactive protein	0.04	0.35
	5.9 Serum total cholesterol	0.06	0.52

### 3.3 Expert positive coefficient and authority coefficient

In the initial phase of the study, one expert opted for nonparticipation in the consultation, culminating in the retrieval of 23 valid questionnaires out of 24 proffered. Notwithstanding, one questionnaire was subsequently adjudicated as invalid, engendering a valid questionnaire recovery rate of 91.7%, and an overall questionnaire recovery rate of 95.8%. In the ensuing second round, 19 valid questionnaires were retrieved from the 22 disseminated, engendering a valid questionnaire recovery rate of 86.4%. Ergo, the questionnaire recall rate surpassed the prescribed threshold of 75.0%, denoting a pronounced level of enthusiasm among the experts.^10^ During the bifurcated rounds of expert consultations, the expert judgment coefficient (Ca) was ascertained at 1.00 ± 0.02 and 0.97 ± 0.06 for the first and second rounds, respectively. Simultaneously, the familiarity coefficient (Cs) was delineated at 0.94 ± 0.11 and 0.85 ± 0.16, while the expert authority coefficient (Cr) was computed at 0.97 ± 0.06 and 0.91 ± 0.09. These statistical evidences implicitly attest to the experts' authoritative stance, cogent representativeness, and robust predictive accuracy concerning the index system. Furthermore, a compendium of 12 and 7 experts proffered recommendations and suggestions, respectively.^11^

### 3.4 Concentration of expert opinion

The scrutinization of the initial consultation round divulged that the mean importance scores for primary indicators oscillated between 4.67 and 4.86, whereas the secondary indicators were dispersed within the range of 3.82–5.00. The standard deviations were cataloged as 0.35–0.63 and 0.00–1.31, respectively, and the full scores extended from 40.90% to 100.00%. In the subsequent round, the mean importance scores for Primary and Secondary indicators ranged from 4.53 to 4.76, and 4.37 to 5.00, respectively, with standard deviations recorded as 0.54–0.75 and 0.00–0.90. The full scores encompassed the range of 52.60%–100.00%.

### 3.5 Degree of expert consensus

The dispersion quotient among the expert consultations was evaluated employing the coordination coefficient (Kendall's *W*) and the coefficient of variation (CV). The coefficient of variation (CV) furnishes insights into the vicissitudes of experts' perspectives concerning each index. In the primary consultation, the CVs for the indexes fluctuated between 0% and 33%, and similarly, in the subsequent consultation, the CVs oscillated between 0% and 20%. The values of Kendall's harmony coefficient (*W*) and the concomitant significance tests mirrored the coherence of the experts' scores across the entirety of the indicators. In this inquiry, the Kendall's *W* values were documented at 0.19 and 0.14 for the two successive rounds, epitomizing an acceptable echelon of concordance among the experts.

### 3.6 Weight determination and consistency test of each indicator

The Analytic Hierarchy Process (AHP) methodology was invoked to compute the normalized weight and combination weight for each terminal indicator (refer to Table 3). An augmented weight is emblematic of an elevated significance for risk assessment. In addition, the consistency ratio (CR) of each restructured judgment matrix was adjudicated to be subsumed under 0.10, denoting a satisfactory degree of consistency across the entire ensemble of judgment matrices.

## 4 DISCUSSION

### 4.1 Content and weight analysis of each indicator

The principal indicators within the index system were apportioned weights in a descending order, as follows: dietary survey (0.32), anthropometric indicators (0.24), medical history (0.19), blood‐biochemical indicators (0.14), and nutrition‐related symptoms (0.11). Such a weight distribution accentuates the preeminence of dietary surveys as the paramount element within the nutrition screening and assessment schema. This schema subsequently extols the merits of physical examinations and medical history, followed seriatim by laboratory determinants and nutrition‐associated manifestations.

### 4.2 Case history

Encompassed within the medical history category, five secondary indicators were discerned. The indicators attributed with heightened weights included: admission symptom score (0.32), age (0.24), clinical stage (0.19), and performance status (0.14). Extant studies have elucidated that nutritional peril or malnutrition in oncological patients is concomitant with factors such as comorbidity, age, clinical stage, mobility, and administered therapies.^6^, ^13^, ^45^, ^46^ It has been empirically substantiated that nutritional risk amplifies by a factor of 1.602 with each successive year of age.^47^ Within clinical praxis, the gravitas of disease scoring is frequently classified utilizing the Nutritional Risk Screening (NRS‐2002) instrument, encompassing 12 disease categories.^48^ Nonetheless, particular disease variants may necessitate an amalgamated assessment of disease acuteness and nutritional requisites, a complex that may engender challenges for clinical professionals and spawn controversy. To augment the precision of nutrition risk screening, the Expert Consensus on Severity of Disease Scoring for Nutrition Risk Screening proffers a more particularized explication of the disease severity score. This is especially pertinent for patients diagnosed with advanced esophageal malignancies, where an integrated treatment regimen, synergizing neoadjuvant therapy with surgical intervention, is prevalently instituted. Beyond surgical treatment or radiotherapy in isolation, the application of chemotherapeutic agents, albeit efficacious against malignancies, may induce collateral impairment to normal tissues, encompassing the gastrointestinal tract mucosa, glands, and gustatory receptors. Such deleterious effects frequently culminate in diminished appetite, nausea, emesis, and other gastrointestinal maladies routinely conjoined with cancer. These symptoms can appreciably inflate the incidence of nutritional risk and malnutrition.^49^, ^50^

### 4.3 Dietary surveys

Dietary surveys, acting as a linchpin in traditional nutritional status evaluation, are envisaged as a relatively autonomous component. Various nutrition assessment instruments, including but not limited to the Patient‐Generated Subjective Global Nutritional Assessment Scale (PG‐SGA) and the Mini Nutritional Assessment Scale (MNA), also assimilate dietary surveys.^51^ Within this dietary survey category, six secondary indicators were identified, the indicators bearing enhanced weights being: significant diminution in food consumption (0.31), quotidian dietary energy intake (valuation) (0.19), quotidian dietary protein intake (valuation) (0.19), and food characteristics (0.14). Reduced alimentary intake is a primary etiology of considerable unintended weight loss in patients, thereby acting as a risk antecedent for malnutrition and a robust prognosticator of mortality within a 30‐day window.^52^, ^53^, ^54^ A comprehensive dietary survey ought not solely to appraise a patient's diminished food intake but should extend to an encompassing examination of overall dietary consumption, to include energy and protein necessities, prevailing intake approximations, and alimentary predilections.^9^

### 4.4 Nutrition impact symptoms

The Nutrition Impact Symptoms (NIS) category subsumes 12 secondary indicators, with the ensuing indicators attributed with greater weights: difficulty in eating/dysphagia/swallowing dysfunction (0.18), choking or coughing while imbibing water (or liquid nourishment) (0.14), and decreased appetite (0.11). Nutrition impact symptoms are emblematic of manifestations that encumber an individual's capacity to ingest aliment, culminating in weight diminution. While expert consultations within this investigation did not evince a marked significance of nutrition impact symptoms in the nutritional screening and assessment process, the author postulates that weight loss is a recurrent phenomenon in esophageal cancer patients throughout the entire perioperative interval, persisting subsequent to discharge. Hence, the early discernment and management of nutrition‐related symptoms are pivotal, a sentiment corroborated by other scholarly investigations.^55^ As remote as the 1990s, Cushman KE advanced symptom management to ameliorate the nutritional condition of cancer patients.^56^ In 2010, Kubrak et al. initially delineated NIS as salient determinants precipitating diminished food intake, weight loss, and functional debilities.^57^ Research has manifested that NIS can prognosticate nutritional status and clinical outcomes.^58^, ^59^ The expressions (signs) engendered by NIS comprise an interlinked succession of deleterious reactions that primarily incite a reduction in appetite and dietary consumption, thereby leading to weight loss and the attenuation of muscle mass, culminating in muscle wasting syndrome. Such states not only escalate the peril of malnutrition but may also segue into refractory cachexia, thereby engendering suboptimal treatment response, diminished survival ratios, protracted hospital residences, augmented overall health care expenditures, and an erosion in the quality of life for patients. Anorexia is perceived as a precursory risk indicator of malnutrition, as appetite diminishes irrespective of weight loss.^11^ The construct of NIS harbors some equivocality owing to the heterogeneity and intricacy of assessment instruments, classification methodologies, and contributory factors, encompassing PG‐SGA, the head and neck symptom checklist (HNSC), the Edmonton symptom assessment system (ESAS), the eating symptoms questionnaire (ESQ) et al., which are routinely employed methodologies for probing NIS.^60^, ^61^, ^62^ Although the quintessential clinical scenario would entail an exhaustive and precise examination and documentation of each symptomatology in patients, the contingencies of health economics circumscribe clinical inquiry to concentrate on the most prevalent symptoms encountered by patients. At present, there exists no consolidated evaluation criterion for NIS in esophageal cancer sufferers. Consequently, this study concentrates on nutrition‐related symptoms correlated with esophageal malignancies and corresponding treatments, such as difficulties in swallowing, postprandial discomfort, coughing during fluid/liquid intake, diminished appetite, nausea, and emesis induced by neoadjuvant therapy, etc., to inaugurate preliminary indicators for nutrition impact symptoms in esophageal cancer patients, which constitute the primary etiologies of energy storage depletion in patients' physiologies.

### 4.5 Anthropometric parameters and history

The Anthropometric Parameters and History category encompasses six secondary indicators, with particular emphasis attributed to the following indicators: unintended weight loss (0.29), body mass index (BMI) (0.20), and triceps skinfold thickness (0.20). Body weight and BMI are rudimentary, yet indispensable constituents in the evaluation of nutritional status. Nevertheless, within the purview of nutritional screening and assessment, the clinical emphasis predominantly gravitates toward manifestations of weight loss.^63^ The AEPEN consensus articulates that individuals afflicted with highly catabolic maladies may undergo weight diminution surpassing 10% of their basal body mass within a temporal framework of 3–6 months, while concurrently sustaining a normative BMI.^64^ In scrutinizing weight loss, it is imperative to integrate considerations not merely of quantity and temporality but also of the constituent elements of weight reduction. A diminution in muscle mass bears more grave implications compared to a reduction in adipose tissue. Sarcopenia is integral to the diagnostic criteria for malnutrition, given that the attrition of muscular strength and mass may precede substantial weight loss and may coexist with obesity, a phenomenon termed sarcopenic obesity. This can result in concomitant occurrences of obesity, weight loss, and muscular atrophy, inclusive of instances observed in esophageal cancer patients.^32^ Triceps skinfold thickness serves as a determinant of subcutaneous fat volume, while upper arm muscle circumference provides insight into muscle protein status. By synergizing these two indicators, precise computations of upper arm muscle circumference can be achieved. Dominant hand grip strength and calf circumference are intricately correlated with nutritional status, specifically in evaluating muscle functionality and protein condition. Recent scholarly explorations have increasingly employed these metrics as facile and readily applicable methodologies for nutritional screening and assessment.^65^, ^66^ The outcomes of expert dialogues within this study reveal that calf circumference did not manifest substantial significance within the nutritional screening and assessment index. However, entities such as the Asian Working Group on Sarcopenia (AWGS) and GLIM criteria endorse its deployment as an unpretentious and pragmatic approach to appraising muscle mass.^67^, ^68^, ^69^

### 4.6 Blood‐biochemical indicators

The Blood‐Biochemical Indicators category is comprised of nine secondary indicators, with particular indicators holding a higher weighting: serum albumin (0.23), serum prealbumin (0.23), and transferrin (0.13). These results resonate with extant scholarly research.^70^, ^71^ However, the scholarly community generally acknowledges that laboratory indicators may augment a multifaceted nutritional assessment. The integration of blood‐biochemical indicators within the nutritional assessment framework persists as a contentious subject, specifically concerning the veracity of hepatic proteins as nutritional diagnostic biomarkers.^72^, ^73^, ^74^

During the inaugural phase of this investigation, a concerted methodology involving extensive literature exploration, collaborative discourse, and meticulous clinical probes was orchestrated to conceptualize a framework for the nutritional screening and assessment indicator system tailored to esophageal cancer patients. This approach served as the bedrock for the subsequent Delphi methodological application. In recognition of the inherent constraints of the Delphi technique, and adhering to extant guidelines and consensus surrounding its execution, deliberate measures were undertaken to fortify quality assurance and bolster the credibility of the Delphi expert consultation outcomes throughout the design and realization of the Delphi investigation. The synthesized framework of the multidimensional nutritional screening and assessment indicator system, meticulously crafted within this inquiry, proffers a robust foundation for a more profound examination of patients' nutritional status and forges an intellectual scaffolding for ensuing clinical corroboration.

### 4.7 Limitations

This study represents a fluid indicator system subject to perpetual refinement. Despite the consensus progressively forged through bi‐round integration of expert perspectives and responses, the resultant Delphi method findings may harbor intrinsic subjectivity and indeterminacy. As such, they ought not to be construed as unequivocal or terminal research conclusions, but rather as contributive references underpinning the theoretical framework of this study. Future endeavors will be directed toward enhancing the evaluative protocols for this indicator system, amalgamating additional feasibility studies and empirical scrutiny to refine and authenticate it, assessing its scientific and clinical applicability, and crystallizing a definitive scholarly stance on the research subject. Moreover, in consonance with the rapid technological evolution, our investigative collective will persistently align its focus with emergent technologies and methodologies to contemporize the indicator system, ensuring concordance with the cutting‐edge clinical praxis and research insights. Through ceaseless inquiry and refinement, we aim to escalate the precision, clinical practicability, and applicability of nutritional screening and assessment, thereby enriching nutritional interventions and educational outreach to patients, and thereby enhancing their overall health status and quality of life.

## AUTHOR CONTRIBUTIONS

**Jingjing Shang:** Conceptualization (equal); data curation (lead); formal analysis (equal); investigation (lead); methodology (equal); project administration (lead); visualization (lead); writing – original draft (lead). **Wen Dong:** Conceptualization (equal); funding acquisition (equal); methodology (equal); writing – review and editing (equal). **Peipei Huang:** Formal analysis (equal); investigation (equal); methodology (equal); supervision (equal); visualization (equal). **Yidan Sun:** Investigation (equal); methodology (equal); supervision (equal). **Yuxin He:** Investigation (equal); methodology (equal); supervision (equal). **Hui Li:** Investigation (equal); methodology (equal); supervision (equal). **Shengwu Liao:** Funding acquisition (equal). **Mei Li:** Conceptualization (equal); data curation (lead); formal analysis (equal); investigation (lead); methodology (equal); project administration (lead); resources (lead); supervision (lead); visualization (lead); writing – original draft (lead); writing – review and editing (lead).

## FUNDING INFORMATION

This study was supported by Guangdong Medical Research Foundation (Grant No. A2022188) and Guangzhou Municipal Science and Technology Program key projects (Grant No. 202103000037).

## Data Availability

The data that support the findings of this study are available from the corresponding author upon reasonable request.

## References

[1] Sung H , Ferlay J , Siegel RL , et al. Global cancer statistics 2020: GLOBOCAN estimates of incidence and mortality worldwide for 36 cancers in 185 countries. CA Cancer J Clin. 2021;71:209‐249. doi:10.3322/caac.21660 33538338

[2] Xia C , Dong X , Li H , et al. Cancer statistics in China and United States, 2022: profiles, trends, and determinants. Chin Med J‐Peking. 2022;135:584‐590. doi:10.1097/CM9.0000000000002108 PMC8920425 35143424

[3] Watanabe M , Otake R , Kozuki R , et al. Recent progress in multidisciplinary treatment for patients with esophageal cancer. Surg Today. 2020;50:12‐20. doi:10.1007/s00595-019-01878-7 31535225 PMC6952324

[4] Hsu P , Chien L , Huang C , et al. Treatment patterns and outcomes in patients with esophageal cancer: An analysis of a multidisciplinary tumor board database. Ann Surg Oncol. 2022;29:572‐585. doi:10.1245/s10434-021-10568-z 34387767

[5] Mao YS , Gao SG , Li Y , et al. Hotspots and prospects of esophageal cancer research in China. Chin J Gastrointes Surg. 2023;26:307‐311. doi:10.3760/cma.j.cn441530-20221222-00535 (in Chinese). 37072305

[6] Cao J , Xu H , Li W , et al. Nutritional assessment and risk factors associated to malnutrition in patients with esophageal cancer. Curr Probl Cancer. 2021;45:100638. doi:10.1016/j.currproblcancer.2020.100638 32829957

[7] Song C , Cao J , Zhang F , et al. Nutritional risk assessment by scored patient‐generated subjective global assessment associated with demographic characteristics in 23,904 common malignant tumors patients. Nutr Cancer. 2019;71:50‐60. doi:10.1080/01635581.2019.1566478 30741002

[8] Wang P , Chen X , Liu Q , Liu X , Li Y . Good performance of the Global Leadership Initiative on Malnutrition criteria for diagnosing and classifying malnutrition in people with esophageal cancer undergoing esophagectomy. Nutrition. 2021;91‐92:111420. doi:10.1016/j.nut.2021.111420 34399403

[9] Movahed S , Varshoee Tabrizi F , Pahlavani N , et al. Comprehensive assessment of nutritional status and nutritional‐related complications in newly diagnosed esophageal cancer patients: a cross‐sectional study. Clin Nutr. 2021;40:4449‐4455. doi:10.1016/j.clnu.2021.01.003 33509666

[10] Molfino A , Imbimbo G , Laviano A . Current screening methods for the risk or presence of malnutrition in cancer patients. Cancer Manag Res. 2022;14:561‐567. doi:10.2147/CMAR.S294105 35210853 PMC8857947

[11] Arends J , Baracos V , Bertz H , et al. ESPEN expert group recommendations for action against cancer‐related malnutrition. Clin Nutr. 2017;36:1187‐1196. doi:10.1016/j.clnu.2017.06.017 28689670

[12] Deftereos I , Kiss N , Isenring E , Carter VM , Yeung JM . A systematic review of the effect of preoperative nutrition support on nutritional status and treatment outcomes in upper gastrointestinal cancer resection. Eur J Surg Oncol. 2020;46:1423‐1434. doi:10.1016/j.ejso.2020.04.008 32336624

[13] Santos IM , Mendes L , Carolino E , Santos CA . Nutritional status, functional status, and quality of life—what is the impact and relationship on cancer patients? Nutr Cancer. 2021;73:2554‐2567. doi:10.1080/01635581.2020.1839520 33121266

[14] CSON Oncology , CSFP Nutrition , NAST Oncologists . Guidelines for nutritional treatment of esophageal cancer patients. Chin J Clin Oncol. 2020;47:1‐6. doi:10.3969/j.issn.1000-8179.2020.01.430 (in Chinese).

[15] Muscaritoli M , Imbimbo G , Jager‐Wittenaar H , et al. Disease‐related malnutrition with inflammation and cachexia. Clin Nutr. 2023;42:1475‐1479. doi:10.1016/j.clnu.2023.05.013 37302879

[16] McGovern J , Dolan RD , Skipworth RJ , Laird BJ , McMillan DC . Cancer cachexia: a nutritional or a systemic inflammatory syndrome? Brit J Cancer. 2022;127:379‐382. doi:10.1038/s41416-022-01826-2 35523879 PMC9073809

[17] Cederholm T , Barazzoni R , Austin P , et al. ESPEN guidelines on definitions and terminology of clinical nutrition. Clin Nutr. 2017;36:49‐64. doi:10.1016/j.clnu.2016.09.004 27642056

[18] Arends J , Strasser F , Gonella S , et al. Cancer cachexia in adult patients: ESMO clinical practice guidelines. ESMO Open. 2021;6:100092. doi:10.1016/j.esmoop.2021.100092 34144781 PMC8233663

[19] Webster JM , Kempen LJAP , Hardy RS , Langen RCJ . Inflammation and skeletal muscle wasting during cachexia. Front Physiol. 2020;11:597675. doi:10.3389/fphys.2020.597675 33329046 PMC7710765

[20] Anker MS , Holcomb R , Muscaritoli M , et al. Orphan disease status of cancer cachexia in the USA and in the European Union: a systematic review. J Cachexia Sarcopenia Muscle. 2019;10:22‐34. doi:10.1002/jcsm.12402 30920776 PMC6438416

[21] Brown LR , Laird BJA , Wigmore SJ , Skipworth RJE . Understanding cancer cachexia and its implications in upper gastrointestinal cancers. Curr Treat Option Oncol. 2022;23:1732‐1747. doi:10.1007/s11864-022-01028-1 PMC9768000 36269458

[22] McMillan DC . Systemic inflammation, nutritional status and survival in patients with cancer. Curr Opin Clin Nutr. 2009;12:223‐226. doi:10.1097/MCO.0b013e32832a7902 19318937

[23] Wang YF , An ZY , Lin DH , Jin WL . Targeting cancer cachexia: molecular mechanisms and clinical study. MedComm. 2022;3:e164. doi:10.1002/mco2.164 36105371 PMC9464063

[24] Xiao Y , Yu D . Tumor microenvironment as a therapeutic target in cancer. Pharmacol Therapeut. 2021;221:107753. doi:10.1016/j.pharmthera.2020.107753 PMC8084948 33259885

[25] de van der Schueren MAE , Keller H , Cederholm T , et al. Global Leadership Initiative on Malnutrition (GLIM): guidance on validation of the operational criteria for the diagnosis of protein‐energy malnutrition in adults. Clin Nutr. 2020;39:2872‐2880. doi:10.1016/j.clnu.2019.12.022 32563597

[26] Balsano R , Kruize Z , Lunardi M , et al. Transforming growth factor‐beta signaling in cancer‐induced cachexia: from molecular pathways to the clinics. Cells‐Basel. 2022;11:2671. doi:10.3390/cells11172671 PMC9454487 36078078

[27] Wu Q , Liu Z , Li B , Liu YE , Wang P . Immunoregulation in cancer‐associated cachexia. J Adv Res. 2023. May 6:S2090‐1232(23)00124‐8. doi:10.1016/j.jare.2023.04.018 37150253

[28] Muthanandam S , Muthu J . Understanding cachexia in head and neck cancer. Asia Pac J Oncol Nurs. 2021;8:527‐538. doi:10.4103/apjon.apjon-2145 34527782 PMC8420913

[29] Diakowska D , Krzystek‐Korpacka M , Markocka‐Maczka K , Diakowski W , Matusiewicz M , Grabowski K . Circulating leptin and inflammatory response in esophageal cancer, esophageal cancer‐related cachexia–anorexia syndrome (CAS) and non‐malignant CAS of the alimentary tract. Cytokine. 2010;51:132‐137. doi:10.1016/j.cyto.2010.05.006 20541434

[30] Fouladiun M , Körner U , Bosaeus I , Daneryd P , Hyltander A , Lundholm KG . Body composition and time course changes in regional distribution of fat and lean tissue in unselected cancer patients on palliative care—correlations with food intake, metabolism, exercise capacity, and hormones. Cancer‐Am Cancer Soc. 2005;103:2189‐2198. doi:10.1002/cncr.21013 15822132

[31] Mannelli M , Gamberi T , Magherini F , et al. The adipokines in cancer cachexia. Int J Mol Sci. 2020;21:4860. doi:10.3390/ijms21144860 32660156 PMC7402301

[32] Anandavadivelan P , Lagergren P . Cachexia in patients with oesophageal cancer. Nat Rev Clin Oncol. 2016;13:185‐198. doi:10.1038/nrclinonc.2015.200 26573424

[33] Mueller C , Compher C , Ellen DM . A.S.P.E.N. Clinical guidelines: Nutrition screening, assessment, and intervention in adults. JPEN J Parenter Enteral Nutr. 2011;35:16‐24. doi:10.1177/0148607110389335 21224430

[34] Taylor BE , McClave SA , Martindale RG , et al. Guidelines for the provision and assessment of nutrition support therapy in the adult critically ill patient. Crit Care Med. 2016;44:390‐438. doi:10.1097/CCM.0000000000001525 26771786

[35] Kondrup J . ESPEN guidelines for nutrition screening 2002. Clin Nutr. 2003;22:415‐421. doi:10.1016/S0261-5614(03)00098-0 12880610

[36] Hui Z , Yang W , Shao‐yu MU , et al. Prevalence of nutritional risks, malnutrition and application of nutritional support rates at one Chongqing teaching hospital. Natl Med J China. 2012;92:3417‐3419. doi:10.3760/cma.j.issn.0376-2491.2012.48.009 (in Chinese). 23327702

[37] Bauer J , Capra S , Ferguson M . Use of the scored patient‐generated subjective global assessment (PG‐SGA) as a nutrition assessment tool in patients with cancer. Eur J Clin Nutr. 2002;56:779‐785. doi:10.1038/sj.ejcn.1601412 12122555

[38] CSONSC . Expert consensus on nutritional therapy for patients with malignant tumors. Chin Clin Oncol. 2012;17:59‐73. doi:10.3969/j.issn.1009-0460.2012.01.013 (in Chinese).

[39] Zhang XN , Jiang ZM , Wu HS , et al. NRS 2002 nutritional risk screening and GLIM step 2 for diagnosis of malnutrition (without FFMI currently). Chin J Clin Nutr. 2020;28:1‐6. doi:10.3760/cma.j.cn115822-20190923-00141 (in Chinese).

[40] Subbiah V . The next generation of evidence‐based medicine. Nat Med. 2023;29:49‐58. doi:10.1038/s41591-022-02160-z 36646803

[41] Hohmann E , Cote MP , Brand JC . Research pearls: expert consensus based evidence using the Delphi method. Arthroscopy. 2018;34:3278‐3282. doi:10.1016/j.arthro.2018.10.004 30509437

[42] Nasa P , Jain R , Juneja D . Delphi methodology in healthcare research: how to decide its appropriateness. World J Methodol. 2021;11:116‐129. doi:10.5662/wjm.v11.i4.116 34322364 PMC8299905

[43] Spranger J , Homberg A , Sonnberger M , Niederberger M . Reporting guidelines for Delphi techniques in health sciences: a methodological review. Z Evid Fortbild Qual Gesundhwes. 2022;172:1‐11. doi:10.1016/j.zefq.2022.04.025 35718726

[44] Niederberger M , Köberich S . Coming to consensus: the Delphi technique. Eur J Cardiovasc Nurs. 2021;20:692‐695. doi:10.1093/eurjcn/zvab059 34245253

[45] Deftereos I , Yeung JMC , Arslan J , et al. Assessment of nutritional status and nutrition impact symptoms in patients undergoing resection for upper gastrointestinal cancer: results from the multi‐centre NOURISH point prevalence study. Nutrients. 2021;13:3349. doi:10.3390/nu13103349 34684353 PMC8539371

[46] Bossi P , Delrio P , Mascheroni A , Zanetti M . The spectrum of malnutrition/cachexia/sarcopenia in oncology according to different cancer types and settings: a narrative review. Nutrients. 2021;13:1980. doi:10.3390/nu13061980 34207529 PMC8226689

[47] Weixian W , Li Z , Fengqin Z , et al. Nutritional risk and associated factors in patients with primary liver cancer. J Nurs Sci. 2018;33:86‐88. doi:10.3870/J.Issn.1001-4152.2018.19.086 (in Chinese).

[48] Pianhong Z , Xian S , Xiaoxu H , et al. Expert consensus on scoring disease severity for nutrition risk screening. Zhejiang Med J. 2022;44:1351‐1355. (in Chinese).

[49] Hirano Y , Konishi T , Kaneko H , et al. Weight loss during neoadjuvant therapy and short‐term outcomes after esophagectomy: a retrospective cohort study. Int J Surg. 2023;109:805‐812. doi:10.1097/JS9.0000000000000311 37010417 PMC10389373

[50] Lyu J , Li T , Xie C , et al. Enteral nutrition in esophageal cancer patients treated with radiotherapy: a Chinese expert consensus 2018. Future Oncol. 2019;15:517‐531. doi:10.2217/fon-2018-0697 30457348

[51] Xu YC , Vincent JI . Clinical measurement properties of malnutrition assessment tools for use with patients in hospitals: a systematic review. Nutr J. 2020;19:106. doi:10.1186/s12937-020-00613-0 32957989 PMC7507822

[52] Serón‐Arbeloa C , Labarta‐Monzón L , Puzo‐Foncillas J , et al. Malnutrition screening and assessment. Nutrients. 2022;14:2392. doi:10.3390/nu14122392 35745121 PMC9228435

[53] MacFarling Meure C , Steer B , Porter J . Interrelationships between dietary outcomes, readmission rates and length of stay in hospitalised oncology patients: a scoping review. Nutrients. 2023;15:400. doi:10.3390/nu15020400 36678271 PMC9865609

[54] Hiesmayr M , Schindler K , Pernicka E , et al. Decreased food intake is a risk factor for mortality in hospitalised patients: The NutritionDay survey 2006. Clin Nutr. 2009;28:484‐491. doi:10.1016/j.clnu.2009.05.013 19573957

[55] Martin L , Muscaritoli M , Bourdel Marchasson I , et al. Diagnostic criteria for cancer cachexia: reduced food intake and inflammation predict weight loss and survival in an international, multi‐cohort analysis. J Cachexia Sarcopenia Muscle. 2021;12:1189‐1202. doi:10.1002/jcsm.12756 34448539 PMC8517347

[56] Cushman KE . Symptom management: a comprehensive approach to increasing nutritional status in the cancer patient. Semin Oncol Nurs. 1986;2:30‐35. doi:10.1016/0749-2081(86)90006-9 3633605

[57] Kubrak C , Olson K , Jha N , et al. Nutrition impact symptoms: key determinants of reduced dietary intake, weight loss, and reduced functional capacity of patients with head and neck cancer before treatment. Head Neck. 2010;32:290‐300. doi:10.1002/hed.21174 19626639

[58] Khorasanchi A , Nemani S , Pandey S , et al. Managing nutrition impact symptoms in cancer cachexia: a case series and mini review. Front Nutr. 2022;9:831934. doi:10.3389/fnut.2022.831934 35308290 PMC8928189

[59] MacLaughlin HL , Twomey J , Saunt R , et al. The nutrition impact symptoms (NIS) score detects malnutrition risk in patients admitted to nephrology wards. J Hum Nutr Diet. 2018;31:683‐688. doi:10.1111/jhn.12553 29578256

[60] Kubrak C , Olson K , Baracos VE . The head and neck symptom checklist©: an instrument to evaluate nutrition impact symptoms effect on energy intake and weight loss. Support Care Cancer. 2013;21:3127‐3136. doi:10.1007/s00520-013-1870-z 23852426

[61] Lindqvist C , Slinde F , Majeed A , Bottai M , Wahlin S . Nutrition impact symptoms are related to malnutrition and quality of life—a cross‐sectional study of patients with chronic liver disease. Clin Nutr. 2020;39:1840‐1848. doi:10.1016/j.clnu.2019.07.024 31427181

[62] Noel CW , Forner D , Chepeha DB , et al. The Edmonton Symptom Assessment System: a narrative review of a standardized symptom assessment tool in head and neck oncology. Oral Oncol. 2021;123:105595. doi:10.1016/j.oraloncology.2021.105595 34775181

[63] García Almeida JM , García García C , Vegas Aguilar IM , Bellido Castañeda V , Bellido Guerrero D . Morphofunctional assessment of patient nutritional status: a global approach. Nutr Hosp. 2021;38:592‐600. doi:10.20960/nh.03378 33749304

[64] White JV , Guenter P , Jensen G , et al. Consensus statement: Academy of Nutrition and Dietetics and American Society for Parenteral and Enteral Nutrition. JPEN J Parenter Enteral Nutr. 2012;36:275‐283. doi:10.1177/0148607112440285 22535923

[65] Crestani MS , Grassi T , Steemburgo T . Methods of nutritional assessment and functional capacity in the identification of unfavorable clinical outcomes in hospitalized patients with cancer: a systematic review. Nutr Rev. 2022;80:786‐811. doi:10.1093/nutrit/nuab090 34850196

[66] Bahat G . Measuring calf circumference: a practical tool to predict skeletal muscle mass via adjustment with BMI. Am J Clin Nutr. 2021;113:1398‐1399. doi:10.1093/ajcn/nqab107 33876186

[67] Chen L , Woo J , Assantachai P , et al. Asian Working Group for Sarcopenia: 2019 consensus update on sarcopenia diagnosis and treatment. J Am Med Dir Assoc. 2020;21:300‐307. doi:10.1016/j.jamda.2019.12.012 32033882

[68] Cederholm T , Jensen GL , Correia MITD , et al. GLIM criteria for the diagnosis of malnutrition—a consensus report from the global clinical nutrition community. Clin Nutr. 2019;38:1‐9. doi:10.1016/j.clnu.2018.08.002 30181091

[69] Evans DC , Corkins MR , Malone A , et al. The use of visceral proteins as nutrition markers: an ASPEN position paper. Nutr Clin Pract. 2021;36:22‐28. doi:10.1002/ncp.10588 33125793

[70] Chiang HC , Lin MY , Lin FC , et al. Transferrin and prealbumin identify esophageal cancer patients with malnutrition and poor prognosis in patients with normal albuminemia: a cohort study. Nutr Cancer. 2022;74:3546‐3555. doi:10.1080/01635581.2022.2079687 35652575

[71] Keller U . Nutritional laboratory markers in malnutrition. J Clin Med. 2019;8:775. doi:10.3390/jcm8060775 31159248 PMC6616535

[72] Evans DC , Guenter P , Jensen G , Malone A . Response to “Low albumin levels should be interpreted, but not ignored”. Nutr Clin Pract. 2021;36:504. doi:10.1002/ncp.10648 33580558

[73] Dioguardi FS . Low plasma albumin levels should be interpreted, but not ignored. Nutr Clin Pract. 2021;36:502‐503. doi:10.1002/ncp.10619 33368587

[74] Portuondo JI , Probstfeld L , Massarweh NN , et al. Malnutrition in elective surgery: how traditional markers might be failing surgeons and patients. Surgery. 2020;168:1144‐1151. doi:10.1016/j.surg.2020.08.012 32919780

